# Omental Infarction due to Omental Torsion

**DOI:** 10.1155/2013/373810

**Published:** 2013-12-02

**Authors:** Hideki Katagiri, Kunpei Honjo, Motomi Nasu, Minoru Fujisawa, Kuniaki Kojima

**Affiliations:** Division of General Surgery, Juntendo University Nerima Hospital, 3-1-10 Takanodai, Nerima-Ku, Tokyo 177-8521, Japan

## Abstract

Omental torsion is a rare cause of acute abdomen and sometimes requires surgery. Recently, we encountered a case of omental torsion diagnosed as omental infarction preoperatively. An 18-year-old male presented to our emergency room with a chief complaint of lower abdominal pain since previous 2 days. Because of his history of Down syndrome, an abdominal examination was very difficult. Plain abdominal computed tomography (CT) suggested omental hernia adhering to the right paracolic gutters. Two days after hospital admission, symptoms did not improve, and contrast-enhanced abdominal CT suggested omental infarction. We performed an emergency surgery. Upon exploration of the abdominal cavity, the greater omentum was found to be twisted four times and adhered to the right paracolic gutters. We performed a partial omentectomy. He was discharged 9 days after the surgery. There was no cause of omental torsion in the abdominal cavity, and he was diagnosed as having idiopathic omental torsion. In cases wherein the cause of acute abdomen cannot be detected, omental torsion should be considered, and abdominal CT could be helpful for the diagnosis.

## 1. Introduction

Omental torsion is a rare cause of acute abdomen and sometimes requires surgery. The most common symptom is right lower abdominal pain; thus, it is often misdiagnosed as acute appendicitis, followed by acute cholecystitis and right-sided diverticulitis [1–4]. Some reports have suggested that abdominal computed tomography (CT) can be used to diagnose omental torsion. However, diagnosing omental torsion preoperatively is difficult [1, 2].

Here, we report a patient diagnosed with omental infarction due to omental torsion by contrast-enhanced abdominal CT before surgery.

## 2. Case Presentation

An 18-year-old male presented to our emergency room with a complaint of lower abdominal pain. Because of his history of Down syndrome, abdominal examination was difficult, and he refused examination, particularly on palpation of the right lower abdomen.

Upon hospital admission, his body temperature was 37.4°C. Laboratory examination revealed a white blood cell (WBC) count of 10,100/*μ*L and C-reactive protein (CRP) level of 1.60 mg/dL. Abdominal X-ray findings were unremarkable, and plain abdominal CT findings suggested an omental hernia adhering to the right paracolic gutters. He was admitted to our hospital for followup. 

**Figure 1:**
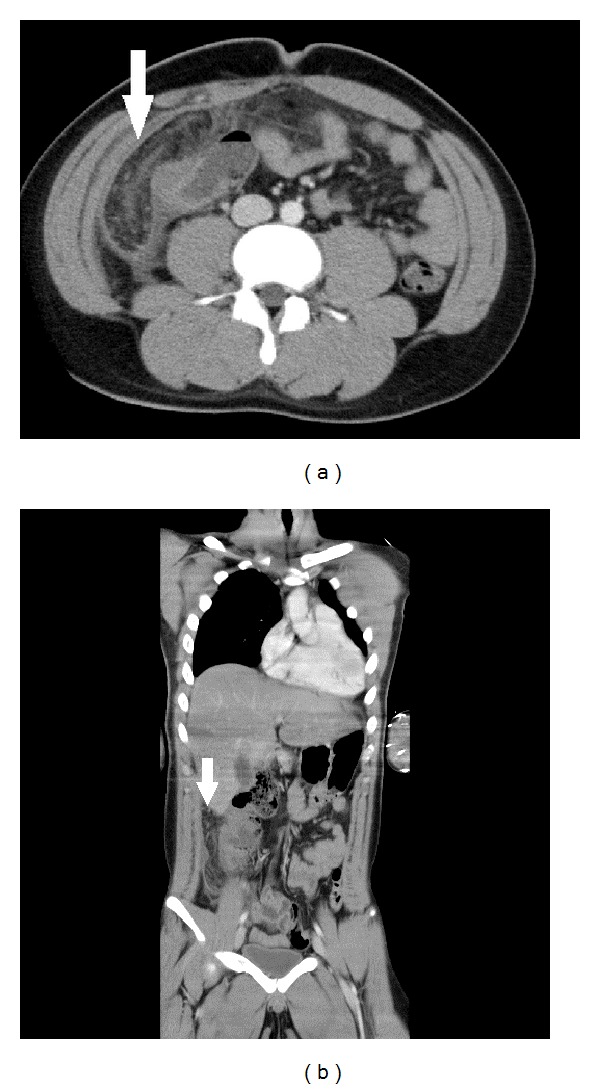
Contrast-enhanced abdominal CT. (a) The fat density of the greater omentum has increased (arrow), a finding that is compatible with a diagnosis of omental infarction. (b) The greater omentum has moved into the right paracolic gutters. A small amount of peritoneal fluid is detected around the omentum.

One day after hospital admission, an increase of CRP levels to 11.67 mg/dL and total bilirubin levels to 2.9 mg/dL was observed. His symptoms did not show improvement, and 2 days after hospital admission these levels did not show improvement and his symptoms worsened. We performed a contrast-enhanced abdominal CT. Elevated fat density of the greater omentum and a small amount of peritoneal fluid around the greater omentum were noted (Figure 1). These findings were compatible with a diagnosis of omental infarction. 

**Figure 2:**
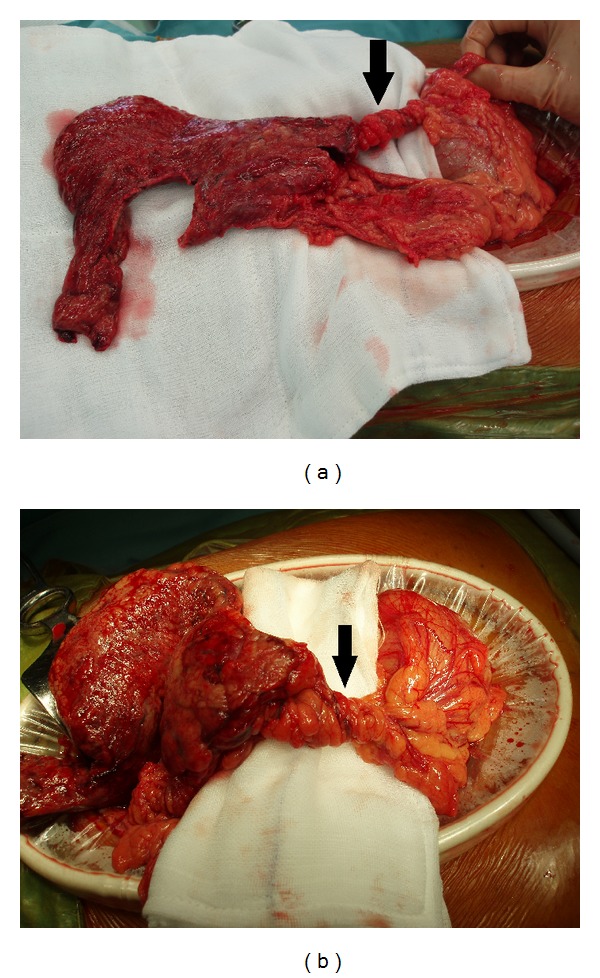
Intraoperative findings. (a) The right part of the greater omentum has twisted four times in a counterclockwise direction (arrow). (b) The distal part of the omentum is infarcted.

We performed emergency surgery with a laparoscopic approach. Upon exploring the peritoneal cavity, the greater omentum adhered strongly to the right side of the abdominal wall, and a small amount of bloody fluid was detected. Completing the laparoscopic approach appeared to be difficult, and thus, we converted to laparotomy. The greater omentum was divided into the left and right parts. The right part of the omentum was twisted four times in a counterclockwise direction, and the peripheral omentum was infarcted (Figure 2). The appendix was slightly swollen, and inflammatory change spread to the cecum. We performed a partial omentectomy and appendectomy. The postoperative course was uneventful, and he was discharged 9 days after the surgery.

A surgical specimen showed intense hemorrhage in the greater omentum and focal inflammatory cell infiltration; appendicitis was not noticed.

## 3. Discussion

Omental torsion causes acute abdominal pain due to the greater omentum being partially or totally twisted and blood flow disturbed [1–3, 5]. The causes of omental torsion are considered to be wide-ranging. Donhauser and Loke suggested a classification of omental torsion by dividing it into “primary” and “secondary” [6]. Omental torsion can also be divided into “unipolar” and “bipolar.” In cases of unipolar omental torsion, the proximal omentum remains fixed, and the other tongues are free. In cases of bipolar omental torsion, the proximal and distal omenta are fixed [4]. Primary omental torsion is always unipolar, and secondary omental torsion develops as unipolar and bipolar. The causes of secondary omental torsion are cysts and tumors in the omentum, internal hernia, and adhesions.

The pathogenesis of primary omental torsion is also considered to be wide-ranging. Adams divided the pathogenesis of primary omental torsion into “predisposing factors” and “precipitating factors” [7]. Predisposing factors include anatomic variations, obesity, and the arrangement of omental blood vessels. Precipitating factors include trauma, hyperperistalsis, and acute changes in body position. Some reports have suggested the precipitating factors of primary omental torsion to be twisting body movements such as in wrestling and horse riding, taking strong purgative drugs, and overeating [1]. In this case, there was no cause of omental torsion and no history that precipitated it; therefore, we diagnosed him as having primary omental torsion.

A major symptom of omental torsion is right-flank and lower abdominal pain; therefore, it is often misdiagnosed as acute appendicitis, followed by cholecystitis and diverticulitis [1–5].The right side of the greater omentum moves more freely than the left side; therefore, omental torsion often develops on the right side. Some cases show a high body temperature but it is usually <38°C, which is considered to be the major difference between omental torsion and acute appendicitis. 

Nishiwaki et al. suggested that in the case of omental torsion, because of hemolysis in the omentum, the total bilirubin level would increase [8]. The total bilirubin in our patient increased to 2.9 mg/dL. Moreover, the long segment of the greater omentum was twisted and infarcted; therefore, hemolysis may have occurred and affected the total bilirubin level.

The preoperative diagnosis of omental torsion is usually difficult. However, some reports have provided images of contrast-enhanced abdominal CT detailing concentric linear strands [1–5, 9]. In our patient, abdominal CT did not show typical images of omental torsion; however, omental infarction was suspected preoperatively upon abdominal CT. In some cases of suspected omental torsion, contrast-enhanced abdominal CT can be helpful for the diagnosis.

 We encountered a rare case of omental infarction due to omental torsion. In some cases, in which the cause of acute abdomen cannot be ascertained, omental torsion should be considered, and abdominal CT could be helpful in the diagnosis.

## References

[1] Scabini S, Rimini E, Massobrio A (2011). Primary omental torsion: a case report. *World Journal of Gastrointestinal Surgery*.

[2] Breunung N, Strauss P (2009). A diagnostic challenge: primary omental torsion and literature review—a case report. *World Journal of Emergency Surgery*.

[3] Doganay S, Gul Y, Kocakoc E (2010). Omental torsion and infarction depicted by ultrasound and computed tomography: an unusual cause of abdominal pain. *Internal Medicine*.

[4] Andreuccetti J, Ceribelli C, Manto O, Chiaretti M, Negro P, Tuscano D (2011). Primary Omental Torsion (POT): a review of literature and case report. *World Journal of Emergency Surgery*.

[5] Sakamoto N, Ohishi T, Kurisu S, Horiguchi H, Arai Y, Sugimura K (2006). Omental torsion. *Radiation Medicine*.

[6] Donhauser JL, Loke D (1954). Primary torsion of omentum: report of six cases. *Archives of Surgery*.

[7] Adams JT (1973). Primary torsion of the omentum. *American Journal of Surgery*.

[8] Nishiwaki S, Yomoda D, Iizuka K, Naitou K, Yoshida M, Ida T (2007). A case of torsion of the omentum secondary to a recurrent inguinal hernia. *Journal of Japan Surgical Society*.

[9] Abdennasser EK, Driss B, Abdellatif D, Mehci A, Souad C, Mohamed B (2008). Omental torsion and infarction: CT appearance. *Internal Medicine*.

